# A health policy and systems approach to addressing the growing burden of noncommunicable diseases in China

**Published:** 2011-06

**Authors:** Kit Yee Chan

**Affiliations:** Nossal Institute for Global Health, University of Melbourne School of Public Health, Peking University Health Sciences Center

Over the past two decades, China has undergone a socio-economic transition unprecedented in human history in terms of its scale and the speed of change (1,2). It is estimated that, while three quarters of the Chinese population lived in rural areas until 1990, almost half were living in cities by 2009 (3). The effect of the one-child policy introduced in 1979 has produced new generations of Chinese children who did not need to share resources with siblings and represent their parents’ sole investment in terms of care, education and mentorship (4,5). This transition period also marked massive government investment in infrastructure, including health investments in access and treatment for all strata of the Chinese population.

My colleagues and I recently documented the dramatic decline in child mortality yielded by these investments, and showed that China has achieved Millennium Development Goal 4 (reduction in child mortality by 2/3 by 2015) nine years ahead of schedule (6). This achievement is all the more significant when one considers the size of China’s population, and the relative performance of other low and middle income countries (7). In population health terms, one of the most remarkable outcomes of China’s extensive transition has been the decrease in maternal and child health problems (8,9).

China’s socio-economic transition will inevitably lead to changes in the health burden of its population, where the fall in maternal and child health burden will soon be replaced by a chronic non-communicable diseases (NCDs) burden in both urban and rural areas. The increase in the burden of non-communicable diseases will be driven by three key factors: (i) a demographic shift marked by an ageing population; (ii) a change from rural to urban ways of life, marked by sedentary lifestyle, high-energy food consumption, increased use of personal transportation, a change in work style, and exposure to outdoor air pollution, and alcohol and tobacco use; (iii) improved access to care and health-seeking behaviour of people in both urban and rural areas (10,11). These factors are already contributing to a rising epidemic of obesity, type 2 diabetes, cardiovascular diseases, degenerative diseases of musculoskeletal system, road traffic accidents, cancers of multiple sites, asthma, depression, dementia and other neuropsychiatric conditions (12-16). Without appropriate inventions, the problem is likely to worsen.

These massive changes and shifts in China’s health burden merit a re-thinking of the future development and structural needs of the Chinese health system. Costs are likely to grow exponentially over the next two decades because of the interaction between an aging population, increased exposures to the key environmental and lifestyle risk factors, and changing patterns of care-seeking and health care expectations. China will soon face millions of cases of diseases such as Alzheimer, and up to a 4-fold increase in the number of new cases of type 2 diabetes, strokes, road traffic accidents and certain types of cancer. China’s health system is currently not structured to efficiently address the management of such a large and growing chronic disease burden. This problem will first become apparent in the fast-growing urban areas, where the majority of Chinese will live, and where population aging and risk factor exposures for NCDs are most immediate.

The most cost-effective strategy for dealing with this growing problem will be to use a combination of strong preventive measures with a strong primary health care system to address the majority of health demands. This would help alleviate the congestion of the hospital services in urban areas, which represent a reasonably strong secondary health care service that must not become over-burdened. Preventive activities will reduce exposure to the main risk factors for chronic non-communicable diseases, and delay or avert a sizeable portion of the burden. This requires a strong public health network in urban areas, and a significant government investment to support the programs. Currently there are small, usually isolated preventive efforts, but the size of the problem will require a coordinated set of preventive programmes at the national-level, which would be implemented in a standardized way in all urban areas.

China currently does not have a system of doctor-maintained, primary care practices comparable to those in the West. At present, in many rural areas, primary health care is carried out by village doctors with relatively low level (Technical College) training which provides them with a licence to treat and prescribe drugs for common illnesses (17). Incentives for medical graduates to work at this basic level of health care are low and few choose this career path. In urban areas, primary health care is provided at community health centres by nurses and medical and/or public health doctors with basic university degrees (18). However, consultation at these clinics for hospital or specialists referrals is not compulsory. Unlike many western health systems, doctors in these clinics do not have an exclusive power of referral to secondary health care. In spite of reforms to the urban primary health care systems, community health clinic positions do not attract the best of medical graduates due to lower salaries, and a lack of prestige and career opportunities.

Patient confidence in the performance of primary care clinics is low, and many prefer to seek treatment in hospitals (19). This has led to long queues in public hospitals, congested with patients better treated in a primary care facility. This will become unsustainable as the NCDs burden increases demand for care. Not enough is being done to develop a stronger primary health care system, which would attract patients away from secondary care hospitals as the point of entry into the health system.

It is widely acknowledged that NCDs can be effectively managed through prevention and primary health care, at a fraction of the cost of hospital care – which is also prone to over-medication and over-treatment. Unless strategies are put in place to reverse the over-reliance on hospitals, the rapidly increasing burden of NCDs will continue to strain the existing health system, and add to the cost of health care, which over time, might interfere with China’s economic growth.

Critical interventions in health system restructuring in China will be needed to improve the cost-effectiveness of the response to the burden of non-communicable diseases in China. Subject to the level of success, China may provide a model for many other low- and middle-income countries which will inevitably face similar problems in future.

I propose that a critical point of intervention in health system restructuring in China, to help address the massive increase in the burden of non-communicable diseases (which will certainly continue over the next two decades), is to:

• Strengthen the network of public health institutes in urban areas, promote them into leading institutions to monitor and evaluate the role of the key risk factors for non-communicable diseases in modern Chinese society;

• Establish sound evidence base on the distribution of common NCDs and their causes for different parts of China and use it for the development of prevention and intervention strategies;

• Implement large-scale national prevention programs that target common chronic non-communicable diseases (eg, cardiovascular diseases, cancer, traffic and workplace accidents) in both urban and rural areas through the network of public health institute. The programs should be based on sound evidence and be monitored and evaluated. Program evaluation should be made available to the public;

• Building upon the National Standards for the Control of Chronic Disease and Prevention set out by the Ministry of Health (China), adopt a holistic approach to prevention that includes strategies to tackle the structural and environmental factors that underlie the risks of chronic diseases (20). The successful implementation of these programmes will necessarily require sound co-ordination with the local media, regulators, health authorities and other stakeholders;

• Strengthen primary health care networks in rural areas by significant government investment into human resources to improve the number of skilled health workers, and target the training of rural doctors towards active participation in disease prevention and patient support in the disease self-management. Lessons should be learned from on-going studies of payment reform (like capitation) as ways of creating provider incentives;

• Strengthen primary health care networks in urban areas by improving the skills and training of the providers, and creating the necessary incentives for medical practitioners to work and remain at these health centres. This will raise the status of the health centres and make them the point of entry into the health system for all but acute care NCDs patients;

• Undertake ongoing case-mix analyses of patient data from secondary care hospitals to (i) determine optimal, cost-effective care strategies, and (ii) identify which categories of patients should be directed to primary care facilities.

• Trial the introduction of a compulsory specialist referral mechanism with primary care facilities as the gate-keepers.

**Figure:**
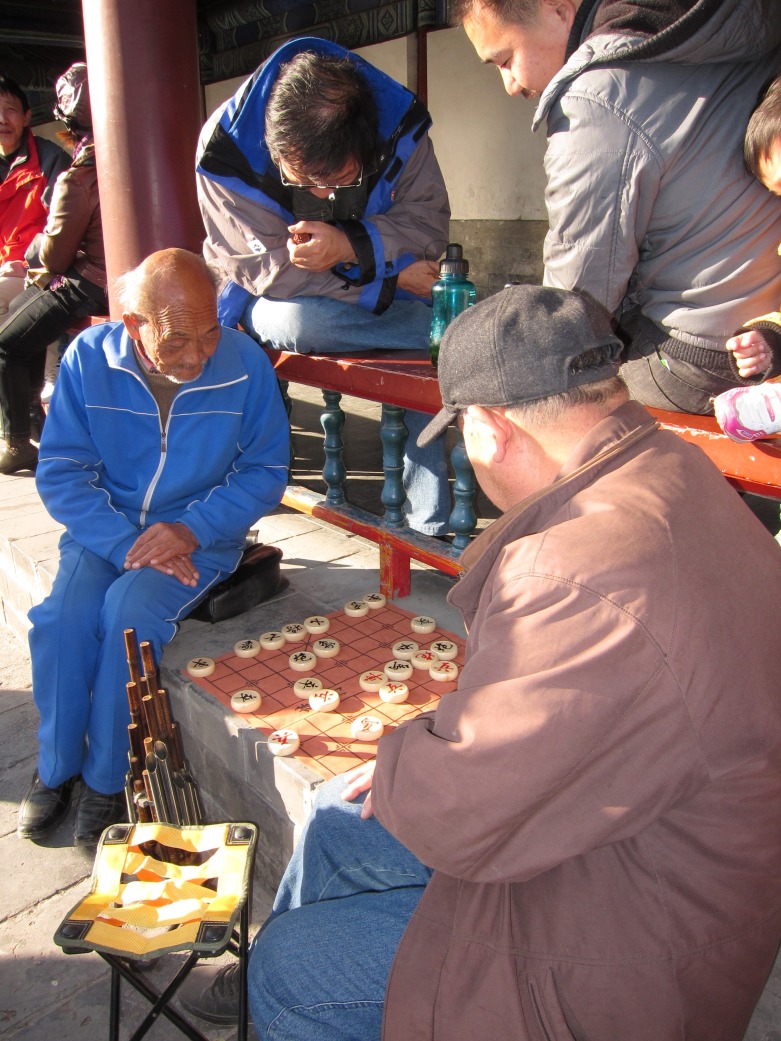
Photo: courtesy of Dr Kit Yee Chan, personal collection

Appropriately implemented, these recommendations, will improve the cost-effectiveness of the response to the growing problem of NCDs in China. Subject to the level of success, this will become a potential model for many other low- and middle-income countries which will inevitably face similar problems, with a slight time delay.
